# Supplementary material for: Mechanisms of A-Type Lamin Targeting to Nuclear Ruptures Are Disrupted in LMNA- and BANF1-Associated Progerias
Source: Cells. 2022 Mar 2;11(5):865. doi: 10.3390/cells11050865 (PMC8909658; doi:10.3390/cells11050865)
Supplement: Supplementary file 1 [file cells-11-00865-s001.zip › Supplementary Figures, Tables and Videos.pdf]

Table S1. Primers used to generate plasmids.

Primer name	Sequence ('5 to 3')
1452	TGGTACGTAGGAATTCGCCACCATGCAGCCTTGGCACG
1453	ATTCCACAGGGTCGACTTACTTGTACAGCTCGTCCATGCCG
1593	TGTGGTGGTACGTAGGAATTCGCCACCATGGTGAGCAAG
1651	CGACTCAGCGGTTTAAACCTACATGATGCTGCAGTT
1833	TGAAAAACACGATAAAGTTTAAAACATGGTGAGCAAGGGC
1834	CACACATTCCACAGGGTCGACTTATCTAGATCCGGTGGA
1873	GCTGTACAAGTCCGGAGAGACCCCGTCCCAG
1874	CATTCCACAGGGTCGACCTACATGATGCTGCAGTT
1903	CTTGCTCACCATGTTTAAACTTTATCGTGTTT
1924	CCAGTGTGGTGGTACGTAGCCACCATGACTACCTCACAGAAGCATCG
1925	CCGGGCCCTCGAATTCTCAGAGGAAAGCGTCACACCA
1927	CGCCGGCCGGATCCGCCACCATGACTACCTCACAGAAGCATCGCGATTTCGTGACAGAACCA ATGGGCGAAAAA
1928	ATTCCACAGGGTCGACTCAGAGGAAAGC
1998	GCTTTCCTCTGAGAATTCGCGGGATCAATTCCG
2004	GTACAAGGACCTCGAGATGACTACCTCACAGAAGCATCG
2005	ATTCCACAGGGTCGACTCAGAGGAAAGC
2035	GACTCAGCGGTTTAAACTTACATAATTGCACA
2036	GACTCAGCGGTTTAAACTCAGCGGCGGCTACCACT
2104	ATCTCGACATCTCGAGAGCCCTACCTCGCAG
2105	CGACTCAGCGGTTTAAACCTAGCGTGCGTGCTGTGA
2181	GCTGTACAAGTCCGGAAGCCCTACCTCGCAG

Table S2. siRNA oligos used in experiments.

Target	Species	Catalog Number	Name	Sequence (5' to 3')
NT control		D-001810-10-05	D-001810-01	UGGUUUACAUGUCGACUAA
			D-001810-02	UGGUUUACAUGUUGUGUGA
			D-001810-03	UGGUUUACAUGUUUUCUGA
			D-001810-04	UGGUUUACAUGUUUUCCUA
LaA/C	Mouse	L-040758-00	J-040758-05	UUAGGGUGAACUUCGGUGG
			J-040758-06	UCAAACUCUCGCUGCUUCC
			J-040758-07	UCUUCAUCGACUUCCUCUA
			J-040758-08	UUCCUCGCUGUAAAUGUUC
BAF	Human	L-011536-02	J-011536-10	UACGACAAUAGCAAUCUUU
			J-011536-11	UUCAUCUUUCUUUAGCACC
			J-011536-12	UGCCACGAAGUCUCGGUGC
			J-011536-13	UUGCCCAGGACUUCACCAA
LEMD2	Human	L-017941-02	J-017941-17	CUAAAAUAUCGGUGGCGAA
			J-017941-18	UCACAGAAGCUGCGACUCU
			J-017941-19	CUGAAUUGGUGACGACUGU
			J-017941-20	CGAGGAGCGGUUACGGGAA
Ankle2	Human	L-181819-00	J-181819-12	AUACUCUCUGAUCCGCUCC
			J-181819-13	UUCCCGAGUUGGUCUGCGG
			J-181819-14	AACGCUACGUCCUAUAUUU
			J-181819-15	AUUCCGAACAACCCAAAUC
Emerin	Human	L-011025-00	J-011025-06	GUAUCCGAAAGAUCUGCGU
			J-011025-07	UACGAGUUGAUCCUACUAC
			J-011025-08	UAGGAUAAUAGGACAGGUC
			J-011025-09	UGAAACAGGGCGGUAGUGC
ZmpSte24	Human	430824	S20066 (sense)	CGAAUAGUUUUGUUUGACAtt
			(antisense)	UGUCAAACAAAACUAUUCGct

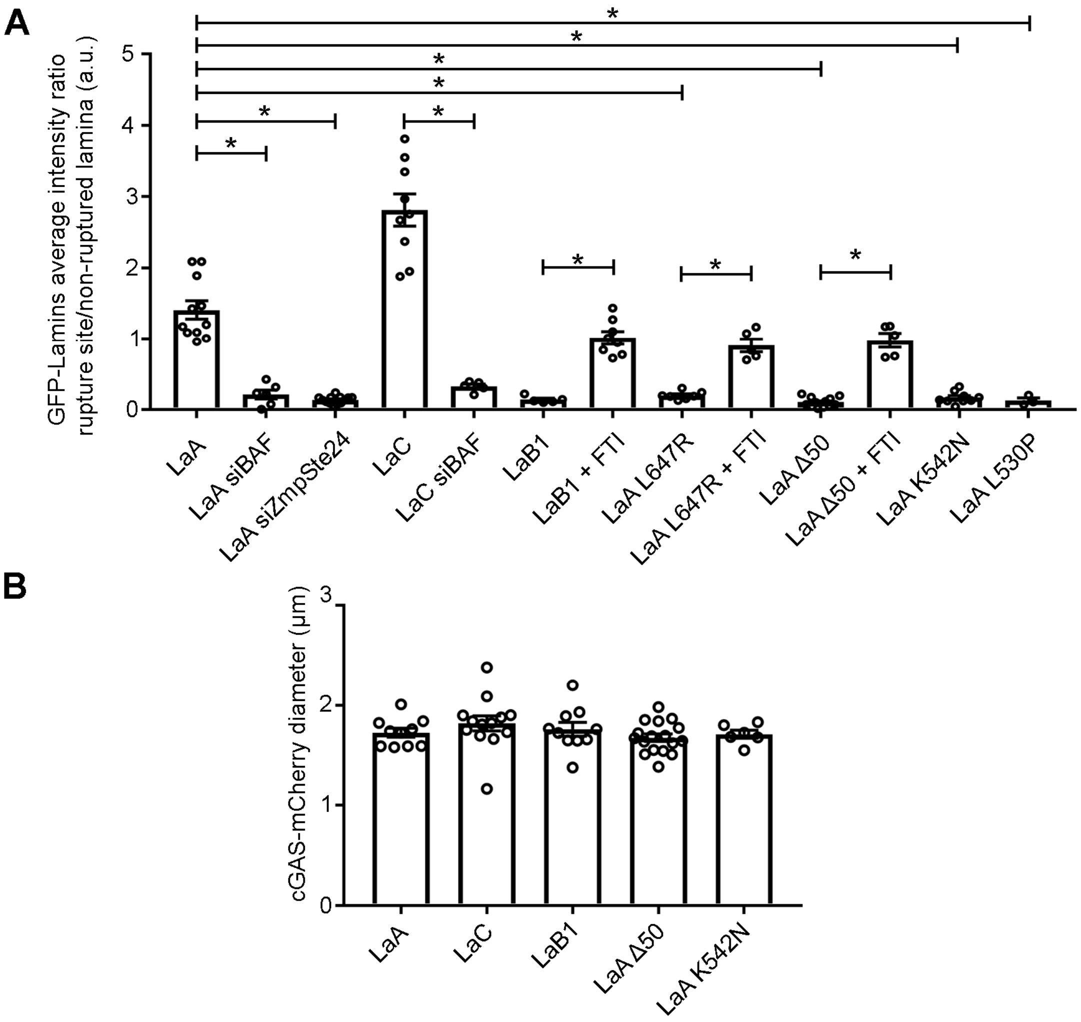

Supplementary Figure S1. **GFP-tagged lamins differentially localize to rupture sites but the ruptures are similarly sized.** (A) Quantification of GFP-tagged lamin average intensity at the rupture site to a non-ruptured lamina opposite of the rupture revealed differing lamin accumulation at ruptures at 5 min post rupture in BJ-5ta cells. The graph represents mean values ± SEM and includes individual values (*n* = 11, 6, 13, 9, 5, 5, 8, 7, 5, 12, 9, and 3 cells respectively; *, $P<0.05$ by a one-way ANOVA with Tukey's post hoc multiple comparison test). (B) To determine rupture hole size at 5 min in our BJ-5ta cells, the diameter of the cGAS-mCherry was measured and graphed. The graph represents mean values ± SEM and includes individual values (*n* = 10, 13, 10, 17, and 6 cells respectively; no significance by a one-way ANOVA with Tukey's post hoc multiple comparison test).

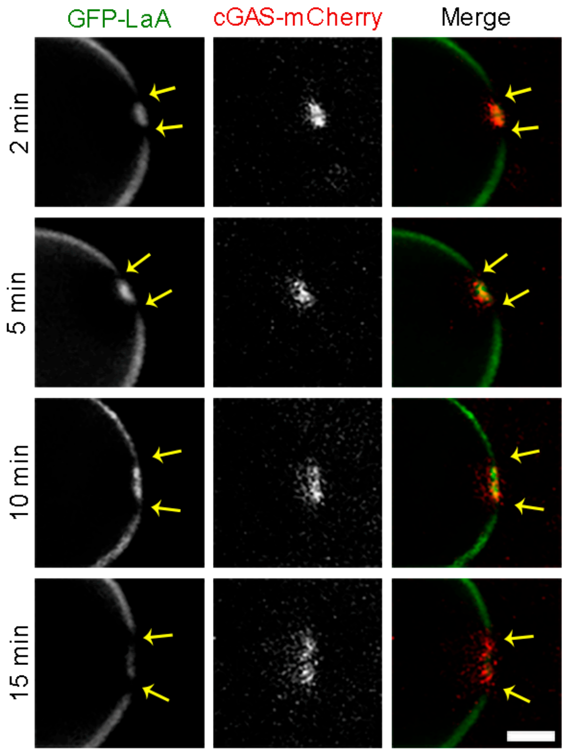

Supplementary Figure S2. **GFP-LaA shows distinct spots of a photobleached lamina on either side adjacent of nuclear ruptures.** Images from Figure 1E with the cGAS-mCherry channel to verify the rupture site boundary. The lamina adjacent of the nuclear rupture remains bleached >15 min post rupture. Scale bar, 2 µm.

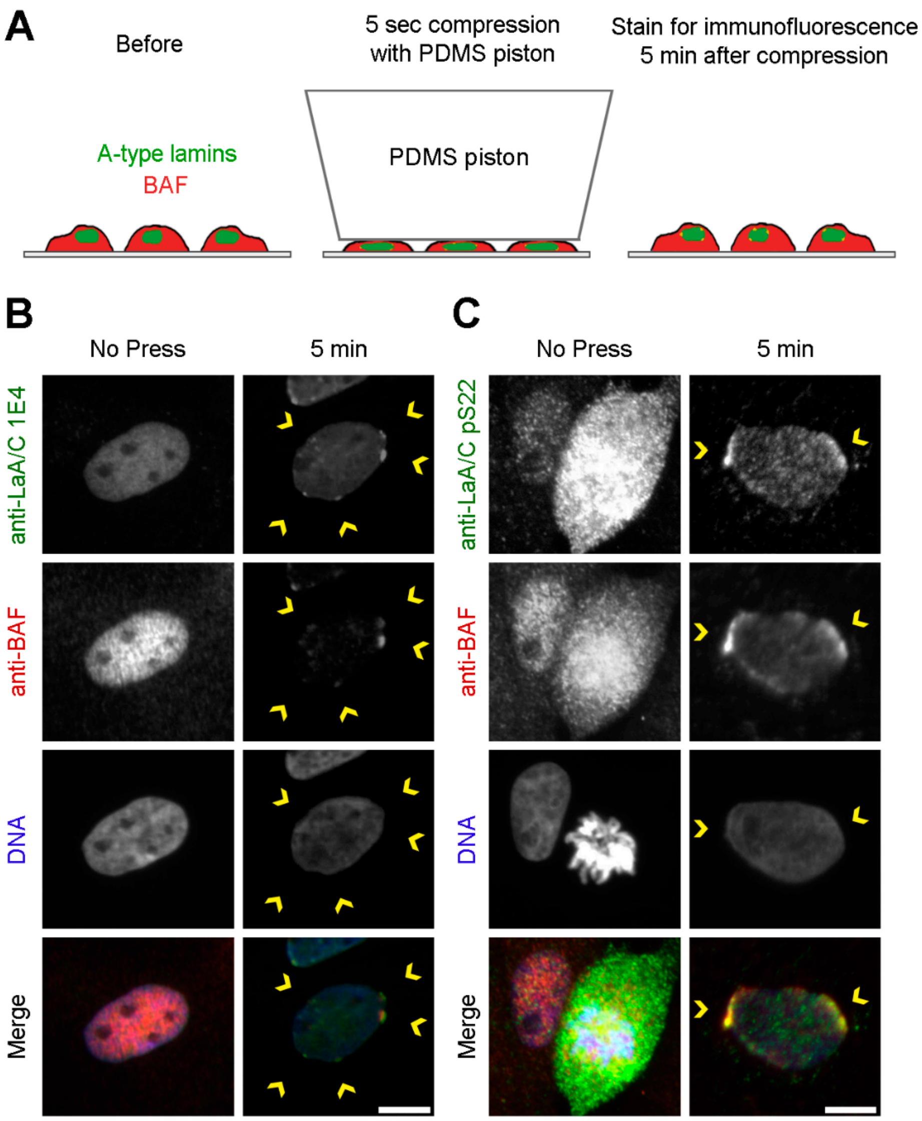

Supplementary Figure S3. **Endogenous nucleoplasmic A-type lamins accumulate at nuclear ruptures**. (A) To induce mechanical nuclear ruptures, cells were grown on coverslips and compressed by a PDMS piston for 5 sec before collection for immunofluorescence. MCF10A cells that experienced mechanically-induced nuclear ruptures via a cell compression chamber were stained to visualize mobile A-type lamins at 5 min post rupture with either (B) the mouse monoclonal antibody 1E4 or (C) an antibody that recognizes A-type lamins phosphorylated at Ser-22. Anti-BAF staining was used as an endogenous rupture reporter. Yellow arrows indicate nuclear rupture locations. Scale bars, 10 µm.

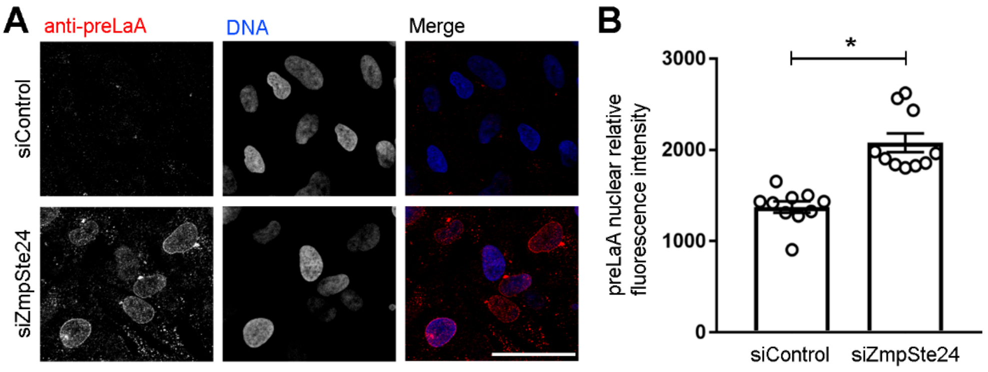

Supplementary Figure S4. Zmpste24 depletion results in prelamin A accumulation in BJ-5ta cells. (A) Immunofluorescence confirmation of endogenous prelamin A (preLaA) accumulation in BJ-5ta cells after 144 hr siRNA knockdown of ZmpSte24. Scale bar, 50 µm. (B) Comparison of endogenous preLaA nuclear relative florescence intensity (RFI) in siControl vs siZmpSte24 siRNA depletion. The graph represents mean values ± SEM and includes individual values (*n* = 10 cells for each condition, *, P<0.0001 by an unpaired t-test).

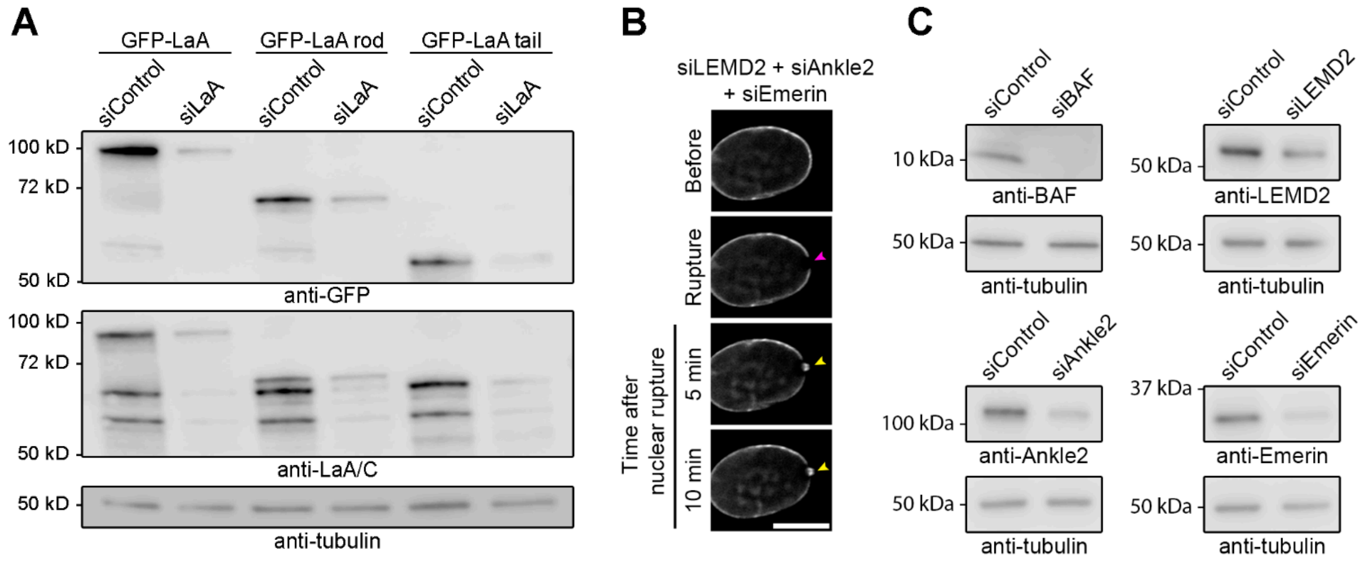

Supplementary Figure S5. The lamin A tail domain is required for lamin A targeting to nuclear rupture sites. (A) Representative immunoblot confirmation of endogenous mouse LaA/C depletion after mouse siLaA/C treatment on NIH3T3 cells stably expressing human GFP-LaA, GFP-LaA rod (aa 1-435), or GFP-LaA tail (aa 391-646). Anti-tubulin was used as a protein loading control. (B) Representative images of laser-induced nuclear rupture events in BJ-5ta cells stably expressing GFP-LaA that underwent a triple siRNA knockdown of the LEM-domain proteins LEMD2, Ankle2, and emerin before induction of nuclear rupture. Scale bar, 10 µm. (C) Representative western blot confirmation of siRNA depletions for BAF, LEMD2, Ankle2, and emerin. Anti-tubulin was used as a protein loading control.

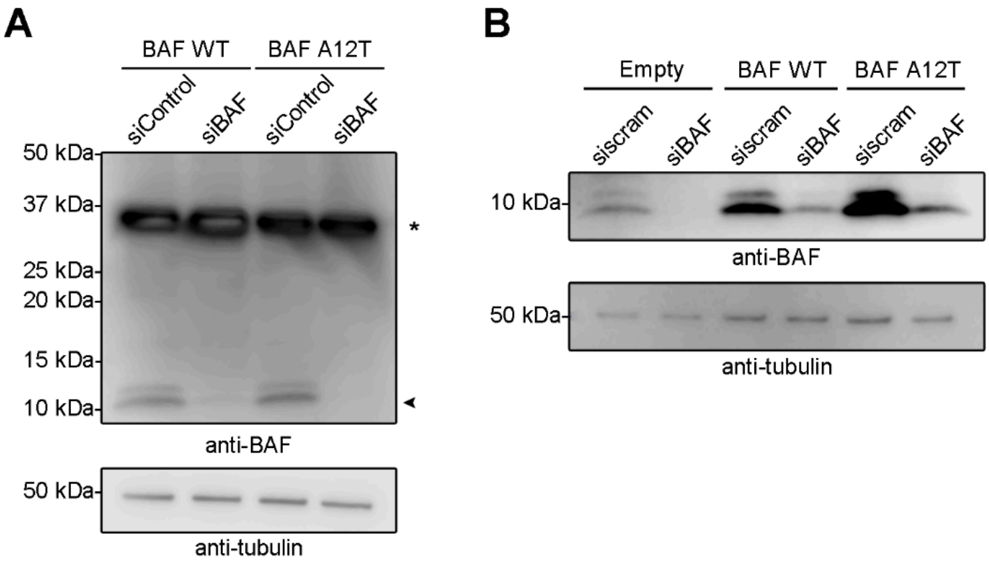

Supplementary Figure S6. SiRNA-resistant BAF is resistant to siRNA BAF depletion. (A) Representative immunoblot confirmation of endogenous BAF depletion after siBAF treatment on BJ-5ta cells stably expressing GFP-tagged codon optimized BAF WT or codon optimized BAF A12T. Asterisk marks GFP-tagged BAF, and arrowhead marks endogenous BAF. Anti-tubulin was used as a protein loading control. (B) Representative immunoblot of siBAF depletion in BJ-5ta cells stably expressing Empty-IRES-GFP-NLS, untagged siRNA-resistant BAF WT-IRES-GFP-NLS, or untagged siRNA-resistant BAF A12T-IRES-GFP-NLS. Anti-tubulin was used as a protein loading control.

Supplementary Video S1. Videos of Figure 1B. BJ-5ta cells co-expressing cGAS-mCherry and GFP-LaA (segment 1), GFP-LaC (segment 2), or GFP-LaB1 (segment 3) were monitored for protein accumulation for 10 min after nuclear rupture (yellow arrowhead). Scale bars, 10 μm.

Supplementary Video S2. Videos of Figure 2A-B. BJ-5ta cells coexpressing GFP-LaA and cGAS-mCherry underwent siControl (segment 1) or siZmpSte24 (segment 2) treatment before laser-induced nuclear rupture and monitored for protein accumulation. (Segment 3) BJ-5ta cells coexpressing GFP-LaA L647R and cGAS-mCherry were monitored for protein accumulation for 10 min after nuclear rupture (yellow arrowhead). Scale bars, 10 μm.

Supplementary Video S3. Videos of Figure 2C-D. BJ-5ta cells coexpressing either GFP-LaA L647R (segment 1) or GFP-LaB1 (segment 2) with cGAS-mCherry underwent laser-induced nuclear rupture after incubation with FTI-277. Protein accumulation was monitored for 10 min after nuclear rupture (yellow arrowhead). Scale bars, 10 μm.

Supplementary Video S4. Videos of Figure 3A. NIH3T3 cells stably expressing human GFP-LaA (segment 1), GFP-LaA rod (aa 1-435) (segment 2), or GFP-LaA tail (aa 391-646) (segment 3) and coexpressing cGAS-mCherry were depleted of endogenous mouse LaA/C before undergoing nuclear rupture and monitored for protein accumulation for 10 min following nuclear rupture (yellow arrowhead). Bars, 10 μm.

Supplementary Video S5. Videos of Figure 4A. BJ-5ta cells coexpressing GFP-LaA and cGAS-mCherry (segment 1), GFP-LaA Δ50 and cGAS-mCherry (segment 2), or GFP-LaA K542N and cGAS-mCherry (segment 3) were monitored for protein accumulation for 15 min following nuclear rupture (yellow arrowhead). Scale bars, 10 μm.

Supplementary Video S6. Videos of Figure 4D. NIH3T3 cells stably expressing human GFP-LaA tail (segment 1), GFP-LaA Δ50 tail (segment 2), or GFP-LaA K542N tail (segment 3) and coexpressing cGAS-mCherry were depleted of endogenous LaA/C before undergoing nuclear rupture and monitored for protein accumulation for 10 min following nuclear rupture (yellow arrowhead). Bars, 10 μm.

Supplementary Video S7. Videos of Figure 5A-B. BJ-5ta cells stably expressing GFP-tagged codon optimized BAF WT (segment 1-2) or BAF A12T (segment 3-4) were depleted of endogenous BAF via siRNA transfection before laser-induced nuclear rupture and monitored for protein accumulation for 10 min after nuclear rupture (yellow arrowhead). Bars, 10 μm.

Supplementary Video S8. Videos of Figure 5G. BJ-5ta cells stably expressing untagged codon optimized BAF WT-IRES-GFP-NLS and either mCherry-LaA (segment 1-2) or mCherry-LaA tail (segment 3-4) were depleted of endogenous BAF via siRNA transfection before laser-induced nuclear rupture and monitored for protein accumulation for 10 min after nuclear rupture (yellow arrowhead). Bars, 10 μm.

Supplementary Video S9. Videos of Figure 5H. BJ-5ta cells stably expressing untagged codon optimized BAF A12T-IRES-GFP-NLS and either mCherry-LaA (segment 1-2) or mCherry-LaA tail (segment 3-4) were depleted of endogenous BAF via siRNA transfection before laser-induced nuclear rupture and monitored for protein accumulation for 10 min after nuclear rupture (yellow arrowhead). Bars, 10 μm.
