# Supplementary material for: Mechanisms of A-Type Lamin Targeting to Nuclear Ruptures Are Disrupted in LMNA- and BANF1-Associated Progerias
Source: Cells. 2022 Mar 2;11(5):865. doi: 10.3390/cells11050865 (PMC8909658; doi:10.3390/cells11050865)
Supplement: Supplementary file 1 [file cells-11-00865-s001.zip › Uncut western blot images.pdf]

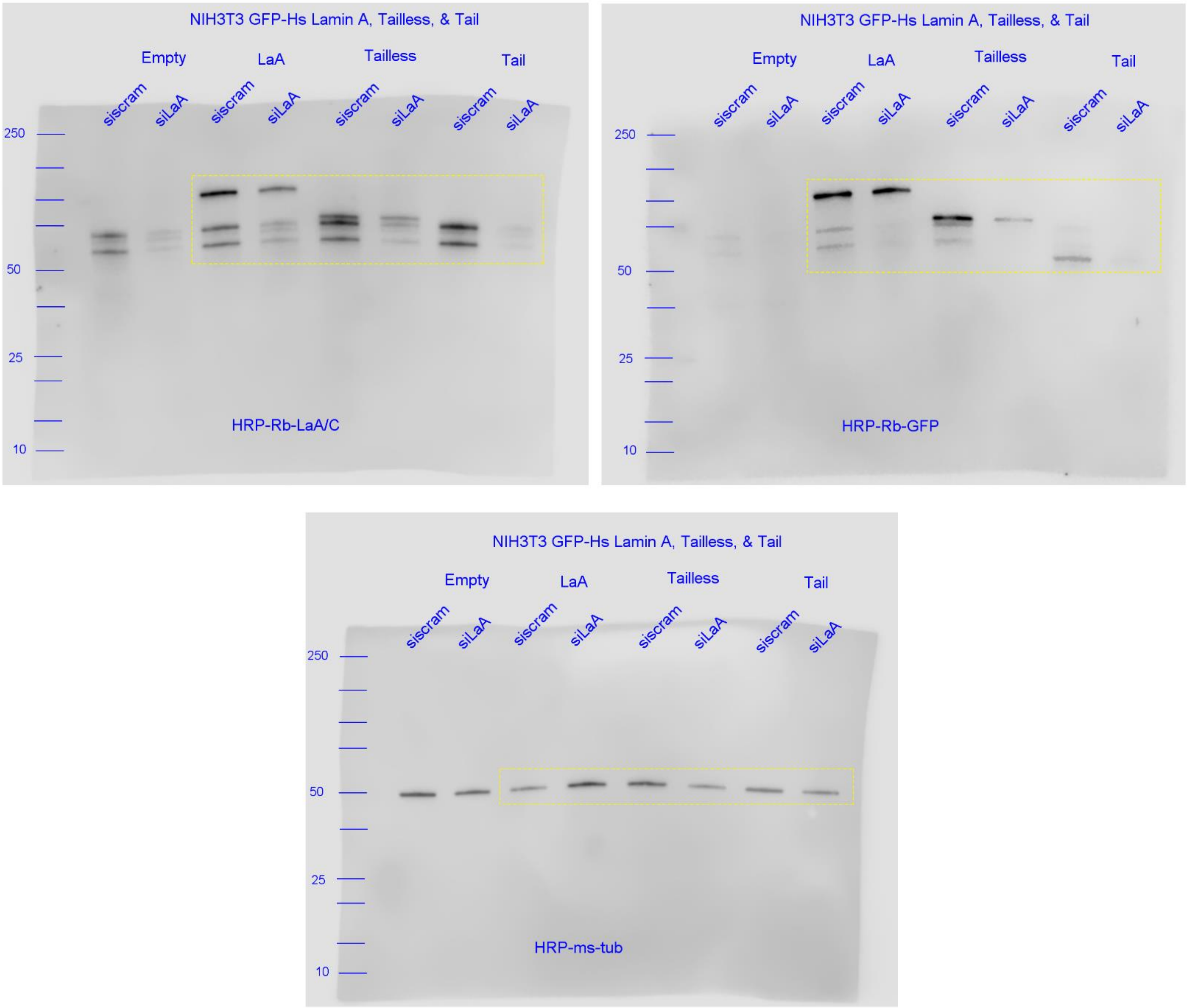

Image 1: Uncut western blots for supplemental figure S5A.

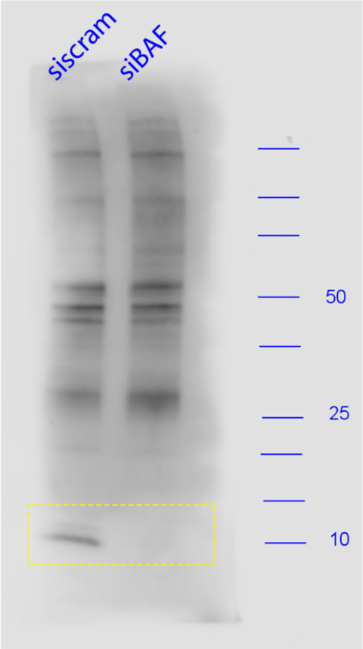

HRP-rb-BAF

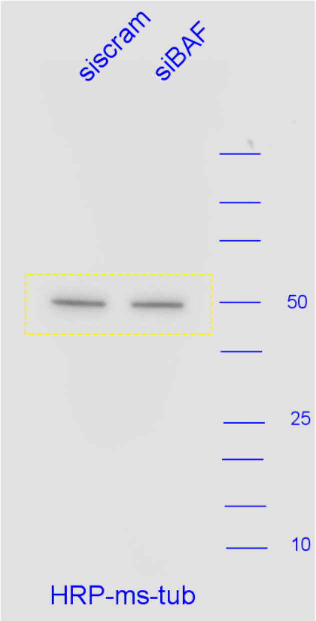

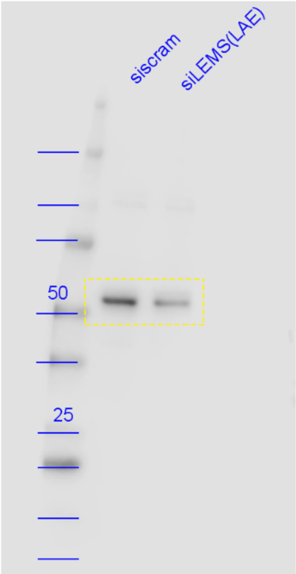

HRP-rb-LEMD2

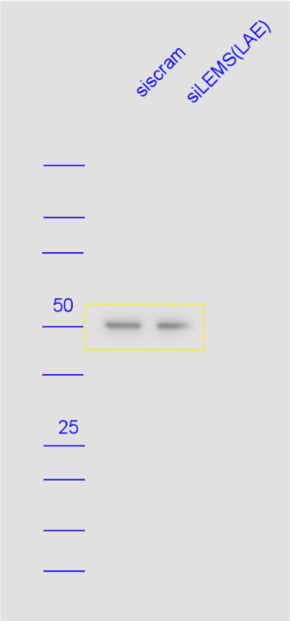

HRP-ms-tubulin

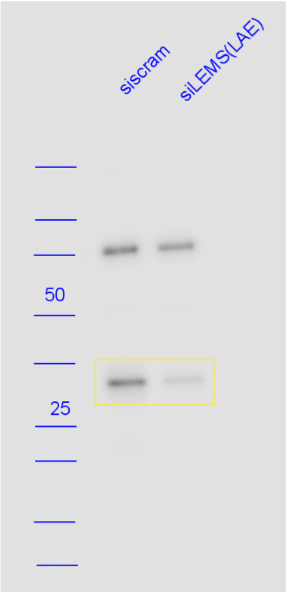

HRP-rb-Emerin

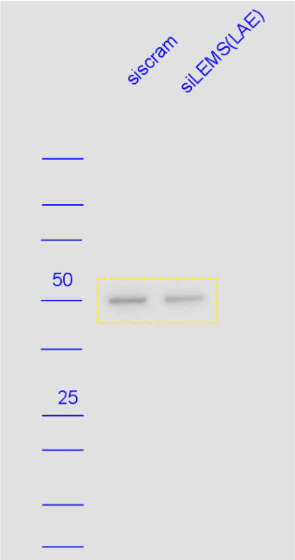

HRP-ms-tubulin

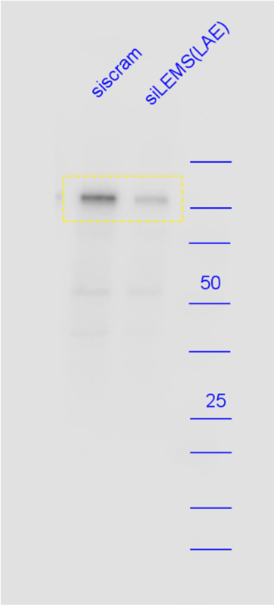

HRP-rb-Ankle2

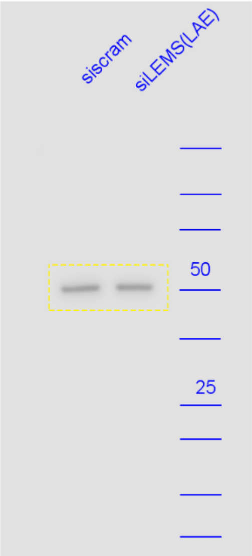

HRP-ms-tubulin

Image 2: Uncut western blots for supplemental figure S5C.

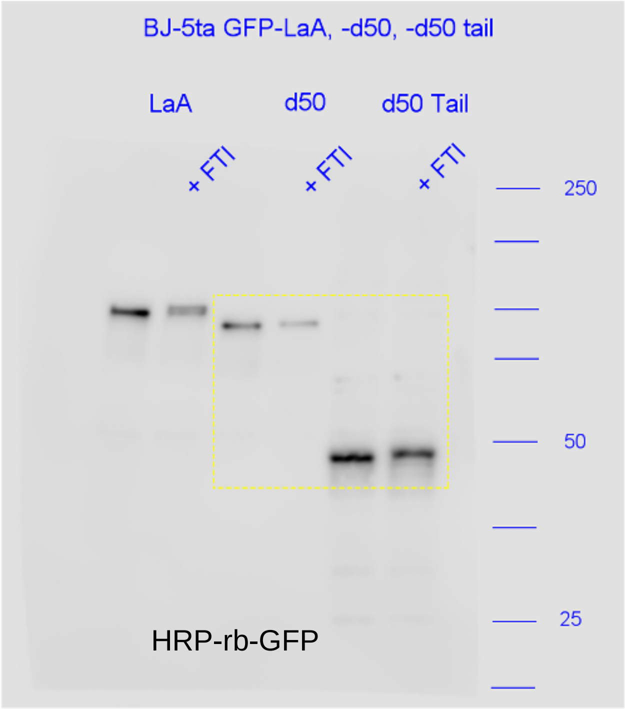

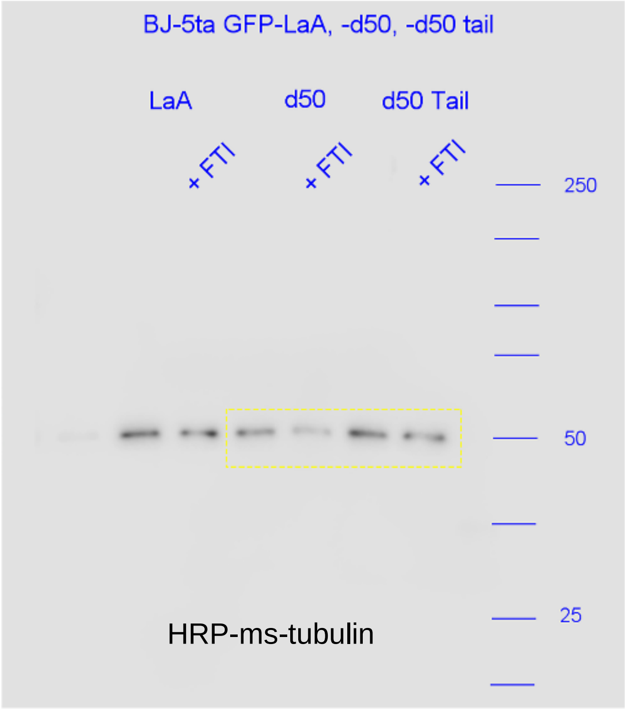

Image 3: Uncut westerns for figure 4E.

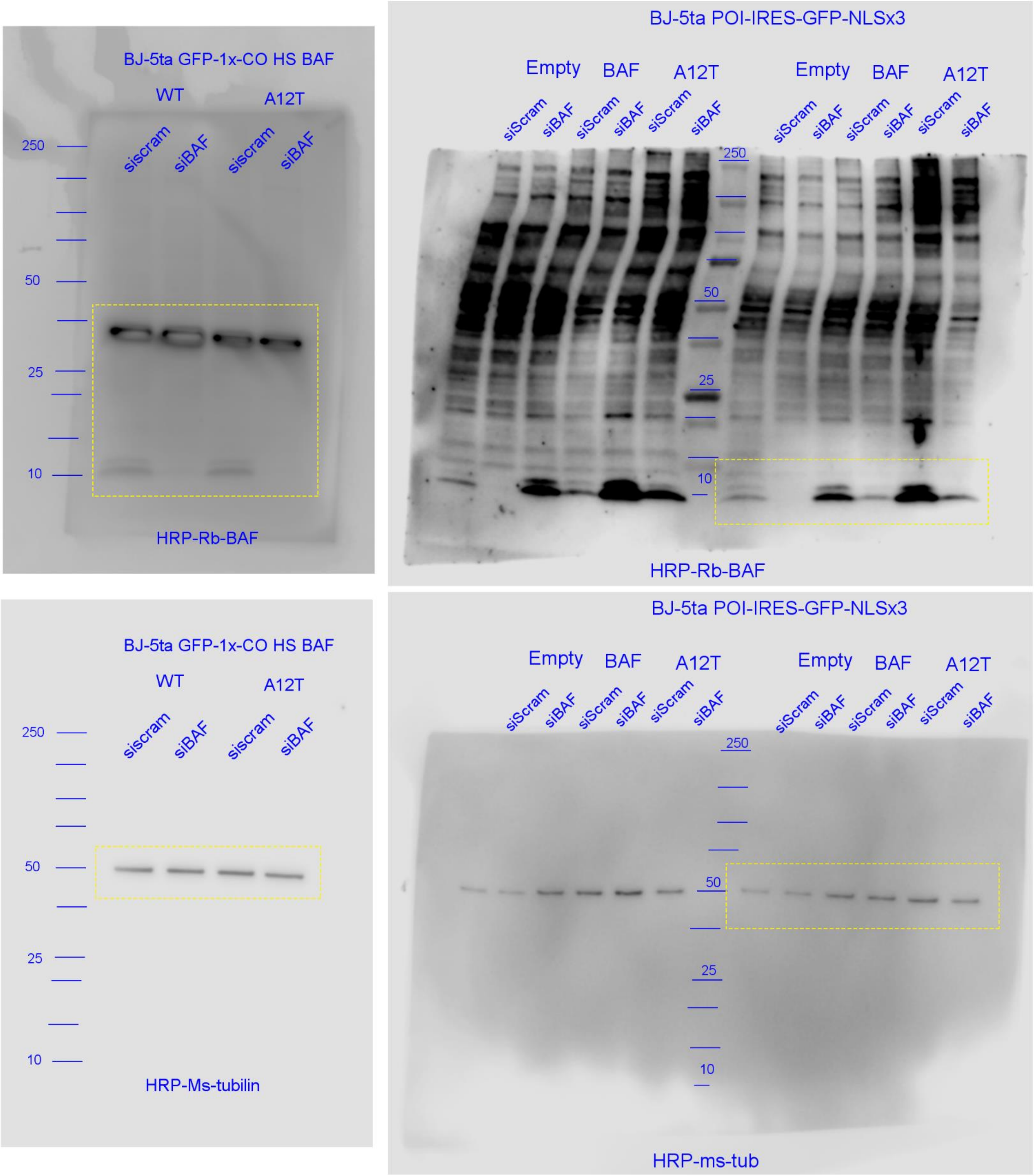

Image 3: Uncut western blots for supplemental figure S6A (left) and S6B (right)
